# Dissertation on the Management of the Mouth and Teeth

**Published:** 1841-09

**Authors:** L. S. Parmly


### ARTICLE II.

*Dissertation on the Management of the Mouth and Teeth.* Read before American Society of Dental Surgeons, at their Annual Meeting in Philadelphia, August 11th, 1841, by L. S. PARMLY, M. D., D. D. S.

MR. PRESIDENT AND GENTLEMEN:—There is no subject which can occupy the mind of man, more full of instruction, or more rich in interest than that which treats of his own organization and preservation, the contemplation of which, while it lays low the pride of the sceptic, is eminently calculated to elevate and ennoble the best feelings of our nature.

It is in reference to public health that I introduce to your attention the care of the mouth and teeth; this branch of education, when properly understood, will supply the deficiencies under which civilized communities have long laboured, and will instruct you as to the means of avoiding and curing diseases of the gums by friction, and of preserving one of the greatest of all earthly blessings, "a sound, clean set of teeth."

If we take a survey of society, constituted as it is, we shall be forced to the conclusion that perfect cleanliness of the mouth and teeth is the last object, on which the attention of parents is fixed, and it is obvious that they have as much power to prevent inflammation of the gums, decay of the teeth and tartar by friction, as physicians have to prevent small-pox by vaccination, and yet how little is public attention called to the care of the mouth, the front door of the temple, not the sepulchre.

There is no truth better understood, and there is none which every day's observation more clearly proves to medical men, than that the loss of the teeth is greatly augmented since the introduction of knives and forks, by which the daily friction upon the enamel has been lessened.*

It is acknowledged on all hands that habit is second nature, therefore the practice should be so modified to the nature and the ability of the individual being, as to produce upon him with uniformity a profitable and beneficial result.

What means can be devised by which an unsuspecting generation may be freely admonished, that there is a noxious fluid contaminating all within its reach? The injury to the enamel is first discovered, and then the decay of the teeth will be in proportion to the age and quantity of mixed putrid matter remaining in contact with the perforated surface, instead of the quality of the teeth as erroneously supposed.

This false doctrine of hereditary weakness appears to have but one sure, unerring remedy, and that is to familiarize mothers, guardians and teachers with the patent apparatus I have invented, kept by most dentists, which will under all circumstances remove the enemy from the mouth and teeth, and in proportion as this truth is promulgated, practised, and duly considered, in the same ratio precisely will they revolutionize their habits to protect the

* It is therefore the duty of parents to furnish children with means perfectly adapted to counteract this fashionable indulgence, so as to render them compatible with the full enjoyment of health, excellence and beauty.

teeth and gums against the attacks of disease, and expose the error of modern philosophy.

The knowledge of using the means alluded to should be possessed by the mother, and society imposes upon her the task of directing the early habits and impressions of childhood.

Now, from what source has the mother received such qualifications? What has society done to impart a knowledge of preventing diseases of the mouth and teeth, and of the physical constitution of that human nature, the care and guidance of which has been entrusted to her? In what part of her education has instruction of this kind been introduced? Why pursue the inquiry, when it is obvious to all that no provision has ever been made to inculcate this important information to her, so indispensable to the welfare of infancy, childhood, mature age, and the evening of life.

There is perhaps no department of human knowledge more neglected than the physical management of youth, who need precept, example, and daily repetition, to enable them to preserve their bodily organs in a cleanly and healthy condition.

If parents would but reflect on the importance of this subject to the future health and happiness of their children, and would avail themselves of the means of obtaining information on a topic so interwoven with the best interests of society, how much good would be dispensed, and how extended the beauty and happiness of mankind!

There is a close connection between happiness and health, between happiness and longevity. Enjoyment is not only one of the ends of life, but it is the only condition of life which is at all compatible with a protracted term of existence.

To suppose that dentition necessarily has its victims, and that the decay and loss of the teeth has no remedy, (or is a matter of necessity,) and that we are without the knowledge and means of checking this dreadful calamity, would be to impugn the most beautiful attribute of heaven's mercy. It is not in keeping with the goodness of God, so to afflict civilized man. Reason bids us suspect, analogy and experience demonstrate that to our neglect is to be attributed this fearful and painful destruction of humanity.

The animal creation around us breathes the same atmosphere, and are subject to the same vicissitudes of season, and carnivorous animals eat similar food to man, and are scarcely if ever tormented by diseases of the mouth and teeth. The true secret of this exemption consists in the peculiar shape and frequent use of their teeth, and the lapping water which effectually removes all accumulations and secretions from the mouth before putrefaction takes place.

The knowledge of preventing decay and irregularity of the teeth is still in its infancy. Such has been the attention given to artificial ones, and the nice description and arrangement of the various productions of the earth, and so profoundly have we explored the arena of nature, that we have had but little time to think of the changes which take place in the mouth, demanding the minutest care, attention and watchfulness.

There is not a bone or muscle of any domestic animal that is not better understood than the teeth of man, who can pursue the concatenations of cause and effect, and embrace in his ken the system of the universe, yet the condition of his mouth and teeth are comparatively neglected, and least cultivated of all human knowledge.

To the philosophic mind it is a subject of keen regret, and indeed it is inconceivable that persons wise, and cautious on all other topics, should manifest such entire indifference in selecting the means of preserving the mouth and teeth from premature disease, pain, deformity and death.

It is my intention to use every proper effort to encourage a desire for knowledge of this kind. We are all directly interested in the spread of truths which, from their very nature, are well calculated to ameliorate the condition of man, and make firm and more lasting that common chain by which we are bound the one to the other.

The dentist who properly appreciates the responsibility he assumes, who feels that on his efforts, skill and judgment may depend the happiness of thousands, enters on the discharge of duty with a zeal and industry worthy his calling, and the great object of his life is to direct his patients and those who have the management of children in the right method of preventing the evils in which neglect and ignorance have involved most of the civilized world, (I mean) a fœted mouth and the causes which produce it, independent of the stomach.

Is the management of the mouth and teeth to be limited to the dental profession? Most assuredly not; it will be found useful to every human being by enabling all to take a rational and daily care of this two-fold operation of the human face divine, as our reasonable service.

The hope of contributing in some small degree to the acquisition of knowledge, which may prepare you to appreciate the important truth that inflammation of the gums, decay and looseness of the teeth are entirely our own fault, by the neglect of that inestimable virtue, perfect cleanliness of the mouth and teeth, which cannot be omitted long, without the infliction of cruel penalties and sacrifices, has induced me to present these remarks, and if so much of health and happiness depend upon the daily observance of this rule, are the proper means within the reach of the great mass of mankind.

This question finds its solution in the contemplation of the mouth and teeth, when viewed as an internal surface, subject to the influence of external agents, and in the preventive means not at hand and under our own control, and which may be transmitted to those you love and to posterity, the richest legacy that man can bequeath:

> "Be wise in time, 'tis folly to delay;
> Cast all your vile cosmetic drugs away;
> Exchange the shallow artifice of dress,
> For nature's more enchanting loveliness;
> And know that blooming health alone abides,
> Where chaste and temperate cleanliness resides."
>
> Dentologie.

Dental science, as connected with health and longevity, has of late become an object of popular attention, and I have met with a few of high endowments who are willing to devote their lives to this department of knowledge, being fully persuaded that they can in no way more efficiently appropriate the treasures they have accumulated than by furnishing and adapting the means to the popular mind in such way as to diffuse among the young people the elements of a science in which all are equally interested, in preventing the annoyances of our senses.

It is unfortunate that authors have not more generally exhibited in a systematic form, a clear and comprehensive view of the phenomena of life, the organization on which those phenomena depend, the necessary influence of physical agents on their production, and the laws, as far as they are understood, on which the action of those agents is based.

If this had been done we should not have to mourn over the ignorance of the community in general, not excepting even the more enlightened and educated classes, with regard to the analysis and shape of the enamel, its diamond texture and unlimited duration by its author, independent of blood or nourishment, the agents which produce decay, and the means by which such agents may be removed from its entire surface daily.

It is thus by pointing out that which has been omitted by others, and inviting your attention exclusively to the enamel of the teeth, which is quite out of the reach of nature's guardian care, and independent of the vital circulation even before it passes through the gums, and destined by its peculiar shape, size and strength to serve as instruments to the animal economy during the longest life. On these facts, I shall hope effectually to demonstrate not only the absurdity, but the positive danger of submitting its beautiful adaptation to chance.

On the management of the infant and his gums will depend his exemption from painful teething and healthy bodily organization and the mental powers as he receives his intellectual and moral aptitudes, taking, as they do, their origin in the predominant states of sensation at a period far earlier in the history of the human being than is generally supposed.

The period of infancy is remarkable for the rapidity with which the enamel is formed and perfected, the abveolar processes are in extreme activity of growth, and the formative predominates over the sentient life. A development of the perceptive organs characterizes this epoch; the brain begins to exhibit greater energy and manifest a wider range of action, the intellectual faculties of the helpless being are in active operation; the power of articulation, an evidence of the increasing energy, now shows itself; a capacity for voluntary locomotion is also acquired, while passion and affection come into play with such constancy and force as to exert over the entire economy of the now irritable and plastic creature a prodigious influence for good or evil.

Now if it be possible to make correct moral perception, feeling, habit, and conduct a part of human nature as any sensation or propensity is, if this be possible for every descendant of Adam without exception, to an extent which would render all more beautiful and consistently virtuous than any are at present, preparation for the accomplishment of this object must be commenced early, as the tender age is susceptible of injurious impressions and dependent upon others for a proper direction of his infant thoughts and the preservation of his teeth and gums, to whom is this high trust generally committed? Should the guidance of childhood and the preservation of the teeth and the mind from the pernicious effects, either of neglect or bad example be left to the result of chance? We think not; common sense condemns the idea.

To train human nature perfect in all its parts, to direct and control the habits and conduct of the child, is one of the most important enterprises in which an individual can engage.

Little has this subject attracted the attention of civilized nations till now. Having been appointed by the American Society of Dental Surgeons to prepare an essay on the proper treatment of the gums and teeth from infancy to age, in order to secure their health and perpetuate their usefulness, I proceed to the arrangement of the subject under the following heads:

1. After more than thirty years experience in dental surgery, and minute observation, I am confident that the gums and teeth of every individual may by proper and daily friction continue in perfect health, independent of all the other organs of the human structure, and fulfil the benevolent designs of nature in masticating the food to the most extreme old age.

2. I consider the inflammation of the gums, the decay and loosening of the teeth as entirely the result of neglect by allowing foreign matter, fluid or solid, to become filthy, corrosive and destructive in quality, and then remaining in contact night and day, producing a chemical decomposition of the enamel before it is polished. This will be a momentous reflection on those who have the care of children, as caries more frequently commence during dentition while the gums are loose, as verified in the decay of the wisdom teeth.

3. Having thus briefly alluded to a great and growing evil in civilized society, and intimated that this evil has its remedy, I shall now exhibit a simple course of treatment which will secure to every son and daughter that commences with their growth, a fair set of healthy teeth and gums, without "spot or blemish," during the natural life.

4. The mouth of the infant should be thoroughly washed with water, not only at birth, but always after sleeping, and with sufficient frequency at other times to prevent thrush, and preserve it from the influence of vitiated saliva, and the secretions of the mouth from passing into the stomach, which would engender worms and cold phlegms.

5. The gums of the infant during the whole period, from its birth to the appearance of all the teeth, should be rubbed by the finger of the mother or nurse many times in the day, for the purpose of giving them a firm texture, which will be easily absorbed by nature up to the scarf skin for the advancing teeth without soreness, pain or inflammation.

6. As soon as the teeth make their appearance, it should be the duty of the mother or nurse to clean them morning and evening with a small brush (and water,) and as they increase in number, floss silk, flax or hemp well waxed, should be regularly passed between them, moving it up and down a little under the gum, not only for the purpose of removing accumulations, but also to give a fine polish to the enamel, which is the natural protection of the bone and beauty of the teeth.

7. If by these means the temporary or changing teeth, only twenty in number, have been cleaned and prevented from decay, and the gums from inflammation, the next in succession are the four permanent molar teeth, or superior grinders, with their pointed, uneven crowns, appearing soon after the fifth year (with more fangs than any other) quite safe for cracking nuts and hard food that requires breaking up for the stomach. This class of double teeth, intended to receive the whole pressure and force of the jaws during the changing of all the front teeth, the most critical epoch in life, therefore the same clean habit should be faithfully continued, as particular daily attention will prevent irreparable mischief, supersede the ordinary services of the dentist, who should inspect the mouth (or some qualified person) once a month, and then the daily care of cleaning it perfectly after the principal meals with the following instruments appear indispensable for comfort and safety. First a stiff brush of a suitable size, used freely over the crowns of the teeth, and moved briskly up and down as may be convenient to give friction to the gums at the same time. Secondly, The floss silk well waxed should be passed up and down on the right and left side of each tooth, a little under the gums, which will remove all accumulations from the interstices and necks of the teeth where the brush cannot reach. Thirdly, The tooth-polisher formed so as to be applied to the surface of the teeth where any discoloration may be seen, and near the gums or edges; the friction to be continued until the enamel is smooth. Fourthly, The double instrument for removing hard tartar and separating the gums during the growth of the teeth so as to make room to polish and clean the enamel thus exposed, and where the ordinary means cannot be applied with good effect. Fifthly, The tongue scraper, of whalebone, (well made) should be used every morning at least to free the surface and edges of the tongue from foul deposites, and discharge the glands and remove all secretions from the mouth which would injure its general health and prove detrimental to the stomach and lungs of the most vigorous temperament at any period of life. If these instructions are properly persevered in, and the means faithfully used, the gums and teeth of every shape and colour may be preserved through life by every class of people, with little expense, in all climates, and the breath from taint, a consummation devoutly to be wished. In conclusion we will remark, that this state of habits and practices would soon be realized in every well regulated family, if such persons would but reflect on the union and conformation of the teeth, gums and alveoli, as the duration of each depends on the healthy condition of all. How just and applicable are the memorable words of Washington to the different organs of the mouth, "United they stand, divided they fall." Now, as inflammation of the gums causes disease and a wasting away of the alveoli, and this absorption loosens the teeth and involves the whole in disease and ruin, unless the proper means are used as directed. By one thought, Columbus found out the way to make an egg stand up, so we, by adding a little patience and practice will soon find out the right way of keeping the teeth, gums, alveoli, and their fibrous adhesions free from disease, which the old school has not been able to accomplish with its philosophy of two sides. Be not discouraged or deceived in this matter; each tooth has four sides and delicate adhesions, therefore frictions must be used daily to insure their union, and then you will find the enamel a time-proof, unchangeable substance in the summer's heat and winter's cold, the variations of diet and medicine even during fever if kept wet in its pure element, saliva; neither will it weaken by the natural wear of time or place, provided there be a full set of teeth, and alike impregnable to decay where daily friction is maintained on the crowns and four sides of each tooth. Interrogate a mother who has been afflicted with diseases of the mouth and teeth, and witnessed the distressing anguish of her children during dentition, she will tell you that the value of the preventive means could not be estimated; that they are beyond all human appreciation. If this knowledge came to us spontaneously, or was an instinct, parents and nurses would be readily excused, but as it is, they should be held responsible for all the accumulations remaining in contact with the teeth and gums of a child more than six hours, which corrodes and taints the mouth during the first and second dentition or period of culture. The omission of this subject from the ordinary courses of education cannot be because it is without interest, nor because nothing worthy of being communicated has been discovered respecting this management, neither can it be that there is a difficulty in the exposition of what is ascertained of error or real utility since the mind is the subject on which all education is intended ultimately to act, and the instrument through which it must effect whatever influence it exerts, hence no man doubts the importance of a true knowledge of the mental phenomena and of the laws by which they are regulated, and the care of the enamel, the casing of the human ivory of which we have the sovereign and unlimited control, invites to practical application and meditation, daily and hourly, of the highest moment in favour of economy, health, beauty, strength and longevity, besides being an auxiliary to every art, science and accomplishment in society. Finally, the opinions I have now suggested, if not sustained, on this important subject, are founded on practical experience. If, then, health can be improved and disease averted by means so easy and simple, if the growth of the body and the enjoyment of life and comfort can be so materially promoted in like manner, if the symmetry of the countenance, the improvement of the voice, and a more complete enunciation can also be effected by means so easily attainable, who would not be tempted to adopt this plan, thereby establishing in their highest perfection, a mind unfettered by constant sources of irritation, and a body impervious to those tortures, which, with all the vigour of electric shocks, are darted through the medium of the nervous system from a disordered mouth to the remotest part of the frame, distressing in infancy, miserable in youth, and mortifying the remainder of life, in spite of all the ligamentum substitutes.

Friends,—the universal knowledge of these facts will render our professional labours less irksome, and will probably increase them four-fold in renewing the teeth and gums from the danger to which they are hourly exposed, where friction has been neglected, the principal cause of the dilapidated condition of the teeth, even among sailors and soldiers, as well as the fashionable and opulent members of society, a predicament so dependent and slavish will certainly rouse this favoured generation to make an effort for personal enjoyment and national renown, and thus achieve a victory over error which will be more important in its results than any other ever has been since history commenced, in affording protection to God's master-work within the reach of all intelligence when ever taught the secrets.

